# Reactive Arthritis as a Consequence of Infection by Clostridium difficile in a Pediatric Patient

**DOI:** 10.7759/cureus.54647

**Published:** 2024-02-21

**Authors:** Natalia M Barron-Cervantes, Jimena Gonzalez-Salido, Jimena Colado-Martinez, Emily Retana-Estrada, Humberto O Bibiano-Escalante

**Affiliations:** 1 School of Medicine, Universidad Panamericana, Mexico City, MEX; 2 School of Medicine, Universidad La Salle, Mexico City, MEX; 3 School of Medicine, Universidad Nacional Autónoma de México, Mexico City, MEX; 4 Pediatric Orthopedics, Fundación Clínica Médica Sur, Mexico City, MEX

**Keywords:** pediatrics, reactive arthritis, pediatric secondary synovitis, pediatric synovitis, clostridium difficile, pediatric reactive arthritis

## Abstract

Reactive arthritis (RA) is the development of a sterile inflammatory arthritis usually associated with a previously known infection, most commonly from the gastrointestinal or urogenital tract. The diagnosis is clinical, based on the presence of acute oligoarticular arthritis of larger joints developing within two to four weeks of the infection. However, in some cases where the infection is not clear, the diagnosis is a challenge, like in the case presented here. We must always rule out past infections as a cause of arthritis by directly asking about the presence of symptomatology associated with it, presented in the past few weeks. It’s important to emphasize that human leukocyte antigen B27 (HLA-B27) should not be used as a diagnostic tool, and it always needs to be correlated with the clinical features. There is no confirmed evidence in the literature that is in favor of prescribing antibiotic therapy during an acute presentation of RA as it usually presents after the infection is cured.

## Introduction

Reactive arthritis (RA) is a sterile inflammatory arthritis presented as a sequela to a past infection. The most common infections associated with it are urogenital and gastrointestinal infections. In this type of arthritis, the microorganism does not enter the joint cavity; that’s why even though it's associated with an infection, it is a sterile synovitis. However, antigens of the triggering pathogen can be detected in the synovial fluid or tissue. The prognosis is usually better for enteric infections compared to urinary infections.

The diagnosis should always be based on the clinical presentation, oligoarticular arthritis of larger joints developing within two to four weeks of the preceding infection. Despite many attempts, no validated universal diagnostic criteria for RA has been developed. During the International Workshop on Reactive Arthritis in Berlin in 1999, a consensus was made to define RA for patients who present with the classical clinical presentation and in whom the preceding infection is caused by a pathogen commonly associated with this condition [1]. The first-line treatment utilizes non-steroidal anti-inflammatory drugs (NSAIDs); other options include intra-articular injections of steroids and disease-modifying anti-rheumatic drugs (DMARDs). Finally, the use of anti-tumor necrosis factor (TNF) biologics represents the most significant change in therapeutic management for these patients. In literature, the use of antibiotic treatment has not shown a superior effect on pain or overall patient symptomatology, except when there is evidence of an active infection for which the use of antibiotics should be prescribed [2].

This report describes the case of a six-year-old male patient who presented with severe pain in the right inguinal region, with no radiation, that increased with movement in a first-level private surgical center in Mexico City. This case is presented to further expand the knowledge about RA in the pediatric population, as well as highlight the importance of evaluating all possible infectious foci in the patient.

## Case presentation

A previously healthy six-year-old male patient presented to the Emergency Room (ER) with severe pain in the right inguinal region, with no radiation, that increased with movement. He was treated externally with an unspecified dose of ibuprofen per oral (PO) with no improvement of his condition. The patient also mentioned mild abdominal pain and tenderness. Past history included a previous visit to the ER four weeks ago where he was diagnosed with post-infectious mesenteric adenitis and was treated with a single dose of ceftriaxone 1 gram intramuscular (IM). Prior to that visit, the parents had an episode of rhinopharyngitis and otitis media, treated with amoxicillin/clavulanic acid 70 mg PO per day for five days, loratadine 5 mg PO per day for five days, and Ibuprofen 100 mg PO every day for four days. No other relevant information was given in his medical history.

Upon admission, physical examination revealed the patient to be conscious, awake, responsive, and with changes in his expression associated with pain. Vital signs were within normal ranges. The abdomen was symmetrical and had no masses nor distention presented, bowel sounds were found normal. The right inguinal region presented moderate to severe pain that increased with ipsilateral leg flexion, limiting ambulation. His laboratory studies showed leukocytosis and neutrophilia, and the rest of the values were within normal limits (Table 1). 

**Table 1:** Laboratory findings. INR: international normalized ratio; BUN: blood urea nitrogen; HDL: high-density lipoprotein

Parameter		Value	Reference values
Hemoglobin		14.7 g/dL	12-18 g/dL
Hematocrit		44.2 %	36-48%
Platelets		296 x 10^3^/uL	150-450 x 10^3^/uL
Leukocyte count		19.15 x 10^3^/uL	4.5-11 x 10^3^/uL
Absolute neutrophils		17.04 x 10^3^/uL	2.5-7 x 10^3^/uL
Absolute lymphocytes		1.53 x 10^3^/uL	1-4 x 10^3^/uL
Absolute monocytes		0.19 x 10^3^/uL	2-4 x 10^3^/uL
Absolute eosinophils		0.2 x 10^3^/uL	3-3.5 x 10^3^/uL
INR		1.32	0.8-1.1
Prothrombin		14.7 seg	9.8-12.5 seg
Thromboplastin		27.5 seg	24.5-32.0 seg
Glucose		92 mg/dL	72-99 mg/dL
Creatinine		0.62 mg/dL	0.31-1.00 mg/dL
Urea		42.8 mg/dL	7.01-16.8 mg/dL
BUN		20 mg/dL	7-22 mg/dL
Uric acid		6 mg/dL	3.5-7.2 mg/dL
Total bilirubin		1.7 mg/dL	0.3-1.0 mg/dL
Direct bilirubin		0.60 mg/dL	0.1-0.3 mg/dL
Indirect bilirubin		1.10 mg/dL	0.3-1.0 mg/dL
Amylase		34 U/L	40-140 U/L
Lipase		8.4 U/L	25 - 120 U/L

**Figure 1:**
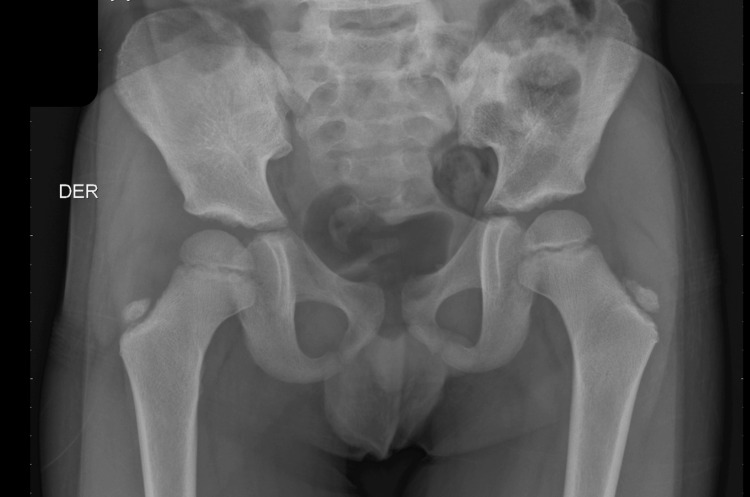
AP bilateral coxofemoral articulation X-ray. Normal calcification expected for age without the presence of fractures, luxations, or any other alterations. DER: right.

**Figure 2:**
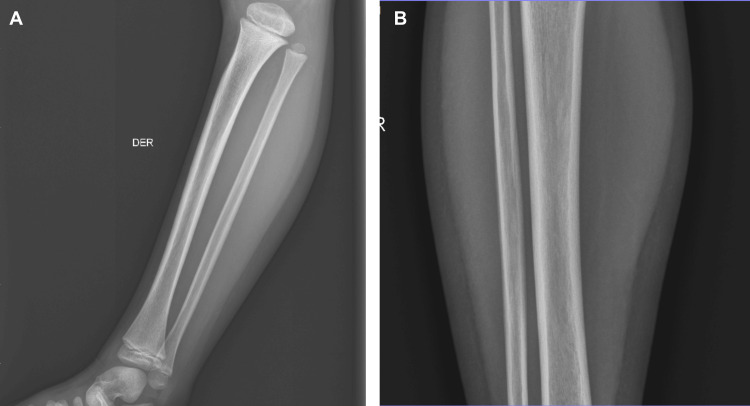
Lateral and AP right leg X-ray. Normal calcification expected for age without the presence of fractures, luxations, or any other alterations. A: lateral view; B: anteroposterior view DER: right

During his stay in the ER, analgesic treatment with acetaminophen 15 mg/kg intravenous (IV) was started. A right anteroposterior (AP) coxofemoral articulation radiography (X-ray) was requested (Figure 1). It reported normal calcification expected for age without the presence of fractures, luxations, or any other alterations. Anterioposterior and lateral radiography of the right leg reported the same findings (Figure 2). 

**Figure 3:**
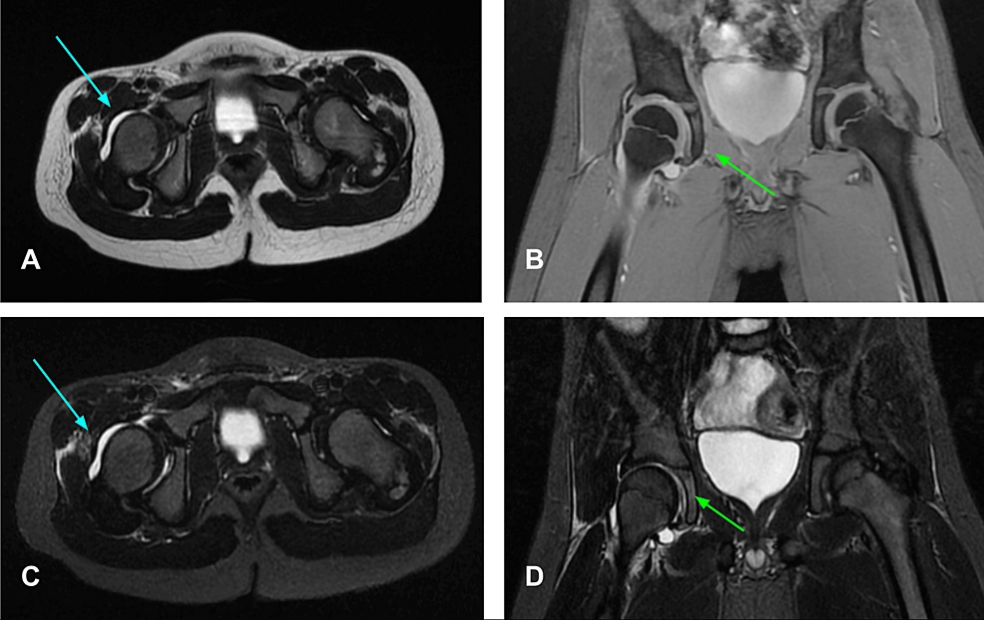
Non-contrasted MRI showing right coxofemoral articulation. Right coxofemoral articulation showing moderate distention of the capsule (blue arrow), the basicervical insertion of the capsule presented bursitis of the adjacent pectineus and obturator externus muscle (green arrow). Left coxofemoral articulation with no alterations reported. Free fluid in the paravesical recesses in the inframesocolic space. (A) Axial plane in T2 weighted image; (B) Coronal plane in Multiple Echo Recombined Gradient Echo (MERGE) sequence; (C) Axial plane in T2 short tau inversion recovery (STIR) image; (D) Coronal plane in T2 STIR image.

As the symptoms persisted, the decision was made to hospitalize the patient. Upon admission to the pediatric floor, a physical examination revealed an increase in pain in the right inguinal region limiting the articular movement almost completely and pain in the left inguinal region developed later. Due to the increase and radiation of pain, magnetic resonance imaging (MRI) was performed which reported moderate distention of the capsule; the basicervical insertion of the capsule showed bursitis of the adjacent pectineus and obturator externus muscle and the left coxofemoral articulation showed no alterations. It was also mentioned that there was presence of free fluid in the paravesical recesses in the inframesocolic space (Figure 3).

**Figure 4:**
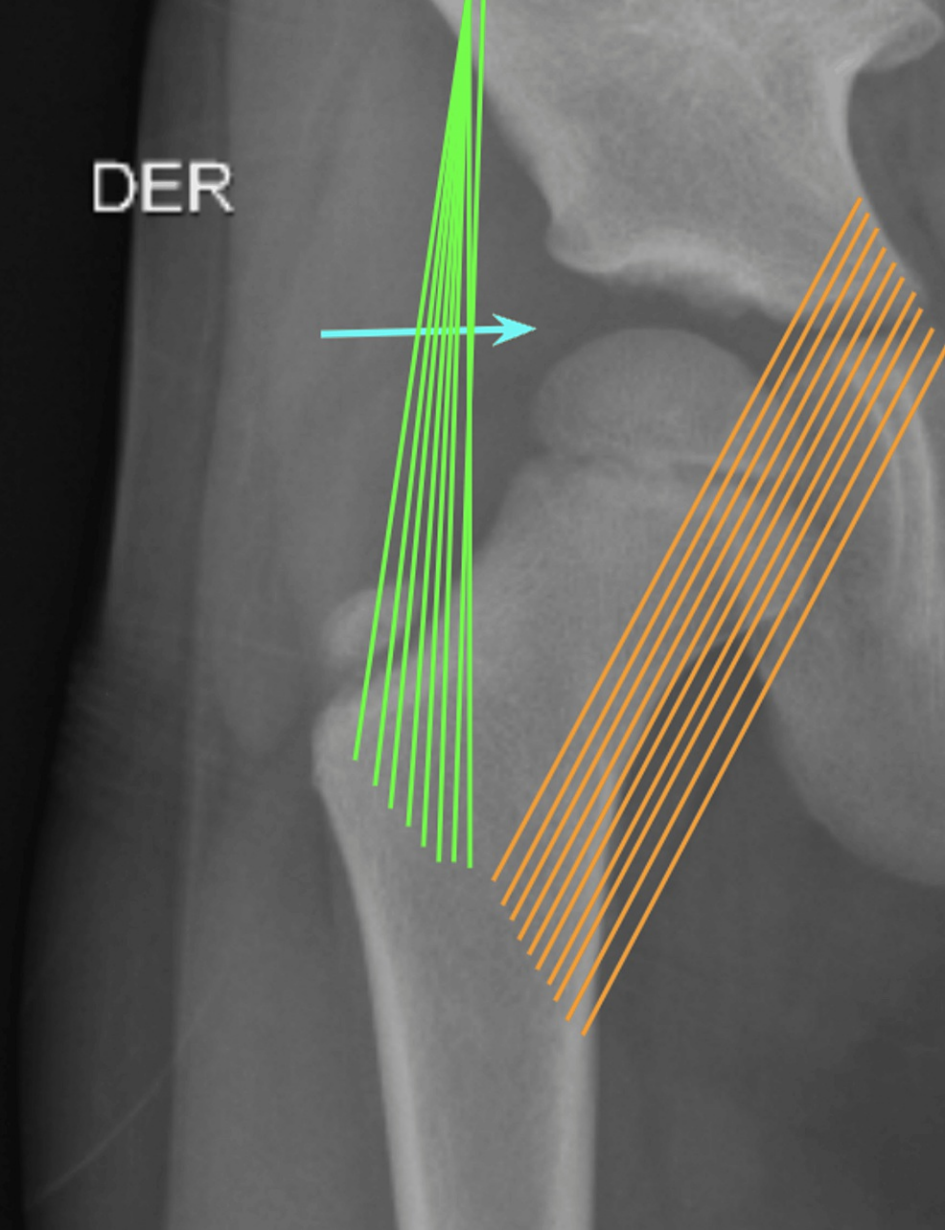
Diagnostic and therapeutic arthrocentesis procedure. Anterolateral arthrocentesis approach under articular traction while the patient was in dorsal recumbent position, which allowed the joint space to open. A nitinol guide and fluoroscopy were employed to ensure adequate entry into the joint (blue arrow). Green lines: Iliofemoral ligament; Orange lines: Pubofemoral ligament.

As a therapeutic and diagnostic approach, an arthrocentesis was performed under sedation in the operating room and the joint fluid obtained was sent for cultures, stains, and pathology. An anterolateral arthrocentesis approach under articular traction while the patient was in a dorsal recumbent position was performed, which allowed the joint space to open by stretching the iliofemoral ligament laterally and the pubofemoral ligament medially. A nitinol guide and fluoroscopy were employed to ensure adequate entry into the joint (Figure 4).

The findings showed that the right coxofemoral sinovial capsule contained 6 mL of serohematic material. Microscopic analysis did not identify microorganisms by Gram or potassium hydroxide (KOH) staining. The patient reported improvement in symptoms, with greater mobility, and a decrease in pain. During his stay, the patient referred to an increase in abdominal pain, accompanied by emesis. Because of this, the pediatric surgery team performed an assessment, ruling out acute abdomen. A CT scan revealed that the colonic tract contained abundant residue, preserving the pattern of the haustras, and a discrete amount of free fluid in the pelvic recess consistent with previous infection. Ruling out bowel obstruction or perforation, no acute abdomen was diagnosed. Because there was no improvement in the abdominal symptoms and the existence of gastroenteritis antecedent, a BioFire® FilmArray® (BioFire Diagnostics, Salt Lake City, Utah, United States) gastrointestinal panel polymerase chain reaction (PCR) was performed. This study showed the presence of *Clostridium difficile*, astrovirus, and sapovirus. Besides, both toxins A and B were negative, but glutamate dehydrogenase (GDH) was positive.

The patient underwent treatment with metronidazole 30 mg/kg/day for his infection. Also, metoclopramide 0.16 mg/kg/dose PO, trimebutine/simethicone 13 mg/kg/day PO, and lactulose 15 ml/day PO were added to treat abdominal distension. The patient was diagnosed with RA in the right coxofemoral joint secondary to *C. difficile *infection. The treatment carried out was polarized light rehabilitation, active movement of lower limbs, acetaminophen 15 mg/kg/dose PO twice a day, and tramadol 0.9 mg/kg/dose PO daily for eight days. After the treatment, he presented a satisfactory clinical improvement, which resulted in the patient being discharged after three days in the hospital for outpatient follow-up. 

## Discussion

RA is a rare clinical presentation, seen in 0.6-27 per 100,000 people per year [1]. As mentioned before, there is no official universal validated diagnostic criteria. The most accepted and utilized system is two major criteria plus one minor criterion from the following: Major criteria include asymmetric, mono, or oligoarticular affection with lower limb involvement and infection preceding arthritis, which may be enteritis or urethritis; Minor criteria involve evidence of a triggering infection, such as positive pathogen test in urine or urethral and cervical swab or enteropathogen-positive stool culture. Also, it should include evidence of a persistent synovial infection, which may include immunohistology testing or a positive PCR test [3].

Even though the course is variable it is usually associated with a favorable prognosis and is self-limited, resolving in three to five months. If arthralgias persist beyond six months, spondyloarthritis should be ruled out by the use of radiology studies. Progression to chronic arthritis, presenting radiographic erosions only presents in 15-30% of all cases and it is associated with the presence of positive human leukocyte antigen B27 (HLA-B27) and a concomitant family history of spondyloarthritis. It is important to mention that the use of HLA-B27 is only beneficial in cases where an autoimmune differential is present, not for all cases of acute RA [2].

Current treatment options vary in between symptomatic treatment while the inflammatory response decreases naturally and options directed at the immunological component of the disease. In pathophysiology, an interplay between the mucosa and gut microbiota, cell subsets, and production of TNF-alpha; IL-23 and Th17 allow for more specific treatment options. Symptomatic rheumatological treatments are mainly NSAIDs, which nowadays are presented as the first-line pharmacological treatment. Then, systemic or intra-articular steroid therapy may be used but there are no controlled studies in the literature that actually support this therapy [2]. A meta-analysis that includes 10 publications proved that there is no superior effect on symptomatology by using antibiotic therapy [4]. However, it is important to emphasize that antibiotic therapy should always be prescribed if there is any data that supports an active infection at the moment. Other options include DMARDs and biological treatments. DMARDs are usually prescribed in cases where arthritis has been persistent for more than three months and has been associated with an autoimmune process. The most commonly used pharmacological options include sulfasalazine and methotrexate. Finally, biological treatments: these drugs have been used since the previous decade. The efficacy is higher and is usually used in cases where all conventional treatments have failed. The anti-TNF agents are the most widely used, which include infliximab and adalimumab [2]. It is important to emphasize that in the majority of cases, there is no need for the use of biologic agents, as analgesic support is enough for patients while the disease takes its natural self-limited course associated with the previous infection.

*C. difficile* is an aerobic gram-positive bacteria, usually existent in normal intestinal flora in 3% of adults and 30-50% of infants. It can be found widely in nature, and spores can be found in soil, water, and feces. It has been reported as a cause of diarrhea caused by the different types of toxins like enterotoxin A and cytotoxin B. *C. difficile* infection has been widely associated with adults, but in the last decade, it has been found as an increasing cause of diarrhea in children. There are several risk factors associated with *C. difficile* infection, mainly hospitalization and administration of antibiotics, but also the use of proton pump inhibitors, prolonged use of nasogastric tube, abdominal surgery, renal failure, immunosuppression, and previous gastrointestinal diseases [5]. In adults, *C. difficile* is the most commonly isolated microorganism related to the use of antibiotics, but it has been increasing in low-risk populations such as children and pregnant women. A large number of patients that are colonized by *C. difficile* have no clinical manifestation. In children, especially those under the age of one, the presence of CD is almost 37% with little infection development. It has not only increased in hospitals but in communities, given the complexity of the pediatric patients that present with it [6]. In pediatrics, 37% of *C. difficile* carriers are infants and 30% are one to six months old babies, related to vaginal membrane rupture, previous use of antibiotics, and environmental conditions. More so, *C. difficile* infection can be transmitted by the hospital personnel, gear, tables, etc. It has diverse manifestations ranging from asymptomatic colonization to mild diarrhea or even fulminant disease characterized by toxic megacolon and sepsis [5].

Furthermore, *C. difficile* and other enteric infections such as *Campylobacter jejuni*, *Salmonella typhimurium*, *and Yersinia enterocolitica* in children have been linked to RA. The physiopathology of RA due to *C. difficile* infection is not fully elucidated; however, it is believed to be an autoimmune response to bacterial antigens that enter the bloodstream via the intestinal mucosa and affect joints and other tissues [7]. 

The primary risk factors include the recent use of antibiotics and proton-pump inhibitors, being 65 years and older, recent stay at a hospital or nursing home, and immunosuppression [8]. Patients who develop RA as a result of *C. difficile* infection typically experience mild diarrhea approximately one to four weeks prior to the onset of arthritis [9]. Moreover, the arthritis typically manifests as migratory polyarthritis affecting primarily the medium and large joints, with a predilection for legs [10]. Diagnostic criteria for RA secondary to CDI were proposed by Putterman and Rainbow. These include the following: Presence of sterile inflammatory arthritis, together with or followed by (i) diarrhea and/or colitis, (ii) previous use of antibiotics, (iii) proof of *C. difficile* or its toxin(s) in stool samples, and (iv) absence of alternative explanation/cause for arthritis and diarrhea [11,12]. Our patient fulfilled the proposed criteria. Treatment consists of analgesics, antibiotic therapy for *C. difficile*, corticosteroids, and DMARDs [10]. Our patient received analgesic medication along with metronidazole, leading to significant clinical improvement.

## Conclusions

RA continues to be a diagnostic challenge today, especially associated with its non-universal diagnostic criteria. In this case, the most important thing to highlight is the infection that triggered everything. It is important not only to rule out the most common urinary and gastrointestinal infections. There is currently very little literature on the association of *C. difficile* with RA. Symptomatic management always has to be the mainstay of therapy, recognizing that if it does not work there are more specific options that can be offered. In this case, the use of antibiotic management is justified since the patient had data consistent with an acute infection that was still active at the time of diagnosis. Active movement and rehabilitation should be seen as an integral part of the treatment in order to avoid atrophy of the limb and subsequent complications associated with weakness or poor joint movement. This case demonstrates that a timely diagnostic approach with personalized management can offer a better prognosis for the patient and improve the associated morbidity.
